# BPDE-induced genotoxicity: relationship between DNA adducts, mutagenicity in the in vitro PIG-A assay, and the transcriptional response to DNA damage in TK6 cells

**DOI:** 10.1007/s00204-017-2003-0

**Published:** 2017-06-07

**Authors:** Ann Liza Piberger, Christopher T. Krüger, Bettina M. Strauch, Beatrice Schneider, Andrea Hartwig

**Affiliations:** Food Chemistry and Toxicology, Institute of Applied Bioscience, Karlsruhe Institute of Technology (KIT), Adenauerring 20a, 76131 Karlsruhe, Germany

**Keywords:** BPDE, DNA adducts, DNA repair, Mutations, Gene expression profiling, High throughput RT-qPCR

## Abstract

Benzo[*a*]pyrene is a known human carcinogen. As underlying mechanism, the induction of stable DNA adducts and mutations have been repeatedly demonstrated. Also, the activation of cellular stress response on the transcriptional level has been described. Nevertheless, the interrelationship between these different events is less well understood, especially at low, for human exposure relevant concentrations. Within the present study, we applied the reactive metabolite benzo[*a*]pyrene diolepoxide (BPDE) in the nanomolar, non-cytotoxic concentration range in human TK6 cells and quantified the induction and repair of stable DNA adducts at the *N* ^2^-position of guanine by HPLC with fluorescence detection. Significant levels of DNA lesions were detected even at the lowest concentration of 10 nM BPDE, with a linear increase up to 50 nM. Relative repair was similar at all damage levels, reaching about 30% after 8 h and 60% after 24 h. Mutation frequencies were quantified as GPI-deficient cells by the recently established in vitro PIG-A mutagenicity assay. Again, a linear dose–response-relationship in the before-mentioned concentration range was observed, also when plotting the number of GPI-deficient cells against the number of DNA adducts. Furthermore, we explored the time- and concentration-dependent DNA damage response on the transcriptional level via a high-throughput RT-qPCR technique by quantifying the impact of BPDE on the transcription of 95 genes comprising DNA damage response, DNA repair factors, oxidative stress response, cell cycle arrest, cell proliferation, and apoptosis. As expected, BPDE activated DNA damage signaling, p53 and AP-1 dependent signaling, oxidative stress response, and apoptosis. However, in contrast to DNA adducts and mutations, the onset of the transcriptional DNA damage response was restricted to higher concentrations, indicating that its respective activations require a certain level of DNA lesions. Altogether, the results indicate that in case of BPDE, DNA lesions and mutations were correlated at all concentrations, suggesting that repair is not complete even at low levels of DNA damage. Considering the ongoing discussion on potential thresholds also for genotoxic carcinogens, the results are of major relevance, both with respect to basic research as well as to risk assessment of chemical carcinogens.

**Electronic supplementary material:**

The online version of this article (doi:10.1007/s00204-017-2003-0) contains supplementary material, which is available to authorized users.

## Introduction

In spite of manifold precautions to reduce exposure towards hazardous chemicals, there are still many carcinogens present in the environment, at workplaces, and in food. They comprise combustion products, carcinogenic metal compounds, carcinogenic organic chemicals, natural bioactive food ingredients as well as carcinogens generated during storage, production, and preparation of food, such as mycotoxins, acrylamide, nitrosamines, and polycyclic aromatic hydrocarbons. Even though exposure levels have dropped considerably during the last decades, this raises the question whether or not there is a carcinogenic risk under realistic exposure conditions. Especially directly genotoxic, i.e., DNA-reactive agents or their DNA-reactive metabolites, are generally assumed to represent risk factors at any concentration, following a linear dose–response also in the low concentration range, implying that even one or a few DNA lesions may result in mutations, and thus, may increase tumor risk. This assumption has repeatedly been challenged during the last years, due to observations that, for example, in case of alkylating compounds such as EMS DNA lesions are linear also in the low dose range, while increases in mutation frequencies follow a non-linear dose–response relationship (Doak et al. 2007; Gocke and Müller 2009; Jenkins et al. 2010; Pottenger et al. 2009; for recent review see Klapacz et al. 2016). This raised the question whether this may apply for all genotoxic substances, due to, for example, complete repair, as well as other DNA damage response systems in cases of low levels of DNA damage, which are overwhelmed in case of higher levels of DNA adducts (Greim and Albertini 2014; Klapacz et al. 2016). Clearly, with respect to a potential impairment of genomic stability, a distinction has to be made between the occurrence of DNA adducts, which may be repaired, and there conversion into mutations, i.e., irreversible alteration of the genetic information. Nevertheless, repair efficiencies in the low dose range may be different in case of DNA lesions generated also endogenously, such as DNA alkylation damage to DNA bases, and lesions induced exclusively or predominantly by environmental mutagens. While cells may be very well adapted to remove oxidatively induced DNA lesions and most types of DNA base damage induced by alkylating agents via base excision repair (BER), many classes of environmental mutagens such as polycyclic aromatic hydrocarbons (PAHs) induce DNA lesions which provoke DNA helix distortions repaired by nucleotide excision repair (NER). As compared to BER, the latter repair pathway is usually slower and not evenly efficient throughout the genome (Fousteri and Mullenders 2008).

Within the present study we addressed the question on dose–response relationships in the low dose range for benzo[*a*]pyrene (BaP)-induced DNA adducts, their repair, the transcriptional DNA damage response, and the induction of mutations in the same cell line, namely TK6 cells. BaP belongs to the group of PAHs formed during incomplete combustion or pyrolysis of organic material. Exposure to BaP provokes manifold adverse effects, including carcinogenicity, immunosuppression, teratogenicity, as well as hormonal effects (Verma et al. 2012). The carcinogenic activity of BaP is attributed to the formation of DNA adducts, resulting from electrophilic attack predominantly at guanine residues by metabolically activated intermediates formed from the parent hydrocarbon. Routes of metabolic activation include the formation of radical cations via P450 and/or peroxidases and the formation of *o*-quinones via dihydrodiol dehydrogenases. For carcinogenicity, the probably most relevant metabolic pathway is connected to the action of cytochromes P450 1A1 and 1B1 and epoxide hydrolase, yielding syn- and anti-BaP-7,8-diol 9,10-epoxides (BPDE), which mainly form adducts at the *N* ^2^ position of guanine. These lesions are substrates of nucleotide excision repair (NER) (Camenisch and Naegeli 2009; Hess et al. 1997). When DNA is replicated prior to their removal, these adducts can lead to mutations and cancer (Melendez-Colon et al. 1999). To exclude cellular detoxification of BaP preceding the induction of DNA lesions, within the present study cells were treated with its DNA reactive metabolite, (+)-anti-benzo[*a*]pyrene 7,8-diol-9,10-epoxide [(+)-anti-BPDE]. Mutations were quantified via the in vitro PIG-A mutagenicity test, which has been recently established for TK6 cells (Krüger et al. 2015). Therefore, to ensure comparable conditions, this cell line was applied for all other endpoints as well. For quantification of (+)-anti-BPDE-induced DNA adducts a highly sensitive HPLC-based assay coupled with fluorescence detection was used, enabling the detection of the respective tetrol I-1 in the very low dose range (Schwerdtle et al. 2002). Finally, we applied a high-throughput RT-qPCR approach to quantitatively elucidate the onset of the transcriptional DNA damage response at the same conditions (Fischer et al. 2016). Our results demonstrate a linear correlation between the amount of DNA adducts and mutations even in the very low concentration range, indicating no threshold-like effect for the conversion of DNA adducts into mutations. Furthermore, the transcriptional DNA damage response was restricted to higher concentrations, at which mutations were already evident.

## Materials and methods

### Materials

RPMI-1640 medium, trypsin, dimethyl sulfoxide (DMSO), and penicillin–streptomycin solutions were purchased from Sigma-Aldrich (Steinheim, Germany) and fetal bovine serum (FBS) from Invitrogen GmbH (Darmstadt, Germany). Cell culture dishes were from Sarstedt (Nuembrecht, Germany). (+)-anti-benzo[*a*]pyrene-7,8-diol 9,10-epoxide ((+)-*anti*-BPDE) was synthesized by Dr. A. Seidel, Biochemisches Institut für Umweltcarcinogene Grimmer (Grosshansdorf, Germany). Mouse anti-human CD55-PE (clone JS11), mouse anti-human CD59-PE (clone p282), mouse anti-human CD19-APC (clone HIB 19), mouse anti-human CD59 (clone p282), goat anti-mouse IgG (polyclonal), and 7-aminoactinomycin D solution (7-AAD, CAS Nr. 7240-37-1) were purchased from Biolegend (London, United Kingdom). Actinomycin D (AD, CAS Nr. 50-76-0) and cycloheximide (CAS Nr. 66-81-9, purity ≥96%) were obtained from Carl Roth (Karlsruhe, Germany). Proaerolysin (CAS Nr. 110616-75-6) was provided by Dr. Peter Howard (University of Saskatchewan, Canada). Cellstar cell culture dishes (for mutant cleansing), 35 × 10 mm, were purchased from Greiner bio-one (Frickenhausen, Germany). All PCR consumables including PCR tubes and strips were obtained from Sarstedt (Nuembrecht, Germany). The primer pairs were synthesized by Eurofins (Ebersberg, Germany) or Fluidigm (San Francisco, USA). DNA suspension buffer, PCR certified water, and TE buffer were obtained from Teknova (Hollister, USA). 2X Assay Loading Reagent and 20X DNA Binding Dye Sample Loading Reagent were purchased from Fluidigm (San Francisco, USA). Bio-Rad (Munich, Germany) provided the 2X SsoFast™ EvaGreen^®^ Supermix with Low ROX. The 2X TaqMan^®^ PreAmp Master Mix was obtained from Applied Biosystems (Darmstadt, Germany) and the exonuclease I from New England Biolabs (Frankfurt am Main, Germany). Consumables for the HPLC-based quantification of BPDE-DNA adducts were obtained from Chromatographie Zubehör Trott (Kriftel, Germany). All other chemicals were purchased from Carl Roth (Karlsruhe, Germany).

### Cell line, culture conditions and treatment with (+)-*anti*-BPDE

Human B-lymphoblastoid TK6 cells were purchased from CLS Cell Lines Service (Eppelheim, Germany). Cells were grown in RPMI-1640 supplemented with 10% heat-inactivated FBS, 0.3 g/L l-glutamine and 2 g/L sodium bicarbonate. Cells were grown at 37 °C and 5% CO_2_ in a humidified incubator and were maintained between 0.08 × 10^6^ and 1.00 × 10^6^ cells/mL. Shortly before the experiment, cells were cleaned from pre-existing mutants as described previously (Krüger et al. 2014). For storage, freshly GPI(−) cleansed TK6 cells were frozen with 3–5 × 10^6^ cells per aliquot in 1 mL FBS with 10% DMSO (v/v).

### Cytotoxicity

Cytotoxicity was determined by loss of colony forming ability. TK6 cells were incubated with (+)-*anti*-BPDE for 1 h, washed with PBS, resuspended in RPMI-1640/FBS, counted, and reseeded in triplicate in 96 well plates with a density of 1.6 cells per well in a total volume of 100 μL RPMI-1640/FBS per well. After 8 days the number of wells with cell growth was counted and cytotoxicity was calculated as % of control.

### Quantification of (+)-*anti*-BPDE-induced DNA adducts

The adducts were quantified upon acidic release as tetrol I-1 using a HPLC/fluorescence assay described previously (Schwerdtle et al. 2002). Briefly, TK6 cells were incubated with increasing concentrations of (+)-*anti*-BPDE for 1 h. After post-incubation times of 0, 8, or 24 h aliquots of each 6.5 × 10^6^ cells of the treated suspension cultures were collected as pellet in Tris-buffered saline (0.0027 M KCl, 0.0513 M NaCl, 0.025 M Tris-base, pH 7.4)/FBS (10%), and washed once with Tris-buffered saline (TBS). After DNA isolation with phenol/chloroform/isoamyl alcohol (25:24:1), the DNA was washed three times with 70% EtOH (Rotisol) and quantified (TECAN NanoQuant). The acidic hydrolysed DNA adducts were quantified as tetrol I-1 by HPLC, equipped with a Dionex UltiMate 3000 pump and a Gynkotek RF 2000 fluorescence detector.

### Pig-A mutation assay

Cell treatment, sample preparation, and analyses of the GPI(−) frequency were performed as described previously (Krüger et al. 2015). After treatment with BPDE for 1 h, TK6 cells were washed and 2 × 10^6^ cells per treatment were antibody-stained in a total volume of 175 µL staining buffer [PBS with 1% BSA (w/v) and 0.1% sodium azide (w/v)]. The cells were incubated with previously pooled mouse anti-human CD19-APC, mouse anti-human CD55-PE, and mouse anti-human CD59-PE for 30 min on ice with an additional centrifugation and resuspension step after 15 min. After antibody treatment, cells were washed twice by adding 1 mL staining buffer and gentle vortexing. Subsequently, cells were incubated with 7-AAD in 500 µL staining buffer for 10 min to exclude dead cells from analysis. Samples were then centrifuged, the supernatant was discarded and cells were fixed in 200 µL fixation buffer [PBS with 1% formaldehyde (v/v) and 2.5 µg/mL AD]. All steps prior to the flow cytometric analysis were performed on ice under exclusion of light. Centrifugation was performed at 250 × *g* for 5 min at 4 °C. Flow cytometric analyses were conducted applying a LSR Fortessa from Becton–Dickinson. Excitation and emission detection of the respective fluorescence dyes were as follows: PE (488–575/26 nm), 7-AAD (488–695/40 nm) and APC (640–670/14 nm). Samples were analyzed with ~8.000 events/s. For one determination of the GPI(−) frequency, 10^6^ GPI(+) cells were collected. To exclude uninformative results of mutagenicity testing due to impaired cell growth after treatment, the relative increase in cell count (RICC) was assessed (Krüger et al. 2015). Directly after the respective treatment, cells were washed with PBS and seeded in triplicate in 24-well plates at a density of 0.1 × 10^6^ cells/mL in a total volume of 500 μL RPMI-1640/FBS per well. After 48-h cell growth, cell numbers were determined and RICC was calculated as described previously:

RICC (% of control) = [(Cell number*_treated_ −0.1 × 10^6^)/(Cell number*_control_ − 0.1 × 10^6^)] × 100, (* Cell number in cells/mL).

### Gene expression analyses

Gene expression analyses via high-throughput RT-qPCR analyses with Fluidigm dynamic arrays on the BioMark™ System were performed as described previously (Fischer et al. 2016). Briefly, 10 × 10^6^ logarithmically growing TK6 cells were treated with BPDE in RPMI containing 10% FCS. After incubation, cells were washed with ice-cold PBS and collected by centrifugation. Total RNA was isolated with MN NucleoSpin^®^ RNA Plus KIT (Macherey–Nagel) according to the manufacturer’s instructions and quantified. 1 µg of total RNA was reverse transcribed in duplicate per sample into first-strand complementary DNA (cDNA) using qScript™ cDNA Synthesis Kit (Quanta) according to the manufacturer’s instructions. Before qPCR, specific target amplification (STA) and exonuclease I (*Escherichia coli*) treatments were performed. A total of 5 µL STA mix was prepared containing 2.5 µL of 2X TaqMan^®^ PreAmp Master Mix, 0.5 µL of the 500 nM pooled primer mixture, 0.75 µL of PCR certified water, and 1.25 µL of cDNA per reaction. STA was performed in a thermal cycler (T100, Bio-Rad Laboratories, Munich, Germany) using the following temperature program: 10 min at 95 °C followed by 12 cycles of 15 s at 95 °C and 4 min at 60 °C and a final holding temperature of 4 °C. Afterwards, 0.4 µL of exonuclease I (Exo I) (initial activity 20 units/µL) was diluted to 4 units/µL with 0.2 µL of 10X Exonuclease I Reaction Buffer and 1.4 µL of PCR certified water per reaction. 2 µL of the exonuclease reaction mixture were added to the STA samples and digestion with Exo I at 4 units/µL was performed according to the following temperature program: 40 min at 37 °C, 15 min at 80 °C, and a final holding temperature at 4 °C. STA and Exo I-treated samples were diluted fivefold with 18 µL of TE buffer. For qPCR, forward and reverse primers (initial concentrations 100 µM) were diluted to 5 µM by adding 2.5 µL of each primer pair to 25 µL of 2X Assay Loading Reagent and 22.5 µL of DNA suspension buffer. For the sample mix, 2.25 µL of STA and Exo I-treated samples were mixed with 2.5 µL of 2X SsoFast™ EvaGreen^®^ Supermix with Low ROX and 0.25 µL of 20X DNA Binding Dye Sample Loading Reagent. Preparation and loading of Fluidigm 96.96 Dynamic Array IFC (integrated fluidic circuit) was performed according to the manufacturer’s instructions. After loading, the chip was transferred into the BioMark™ System (Fluidigm, San Francisco, USA) and qPCR as well as melting curve analysis were performed by running the following temperature program: 2400 s at 70 °C and 30 s at 60 °C, followed by a hot start for 60 s at 95 °C, 30 PCR cycles of 5 s at 96 °C for denaturation and 20 s at 60 °C for annealing and elongation. The melting curve analysis consisted of 3 s at 60 °C followed by heating up to 95 °C with a ramp rate of 1 °C/3 s. Data analysis and depiction was accomplished with Fluidigm Real-Time PCR Analysis and with GenEx software. For normalization, five potential reference genes were available (*ACTB*, *B2* *M*, *GAPDH*, *GUSB*, and *HPRT1*). Finally, potential alterations of the transcript levels of the target genes under investigation were displayed as fold change compared to a control group by calculating relative quantities corresponding to the ΔΔ*C*_q_ method (Livak and Schmittgen 2001).

#### Statistics

For statistical analyses of the data, differences between control and treated samples were analyzed by one-way analysis of variance (ANOVA) followed by the appropriate post hoc-test. The data were tested for homogeneity of variances via Levene test and two-sided Dunnett’s *T* test was used in case of homogeneity, whereas a two-sided Dunnett’s *T3* test was applied upon variance inhomogeneity.

## Results

### Cytotoxicity

**Fig. 1:**
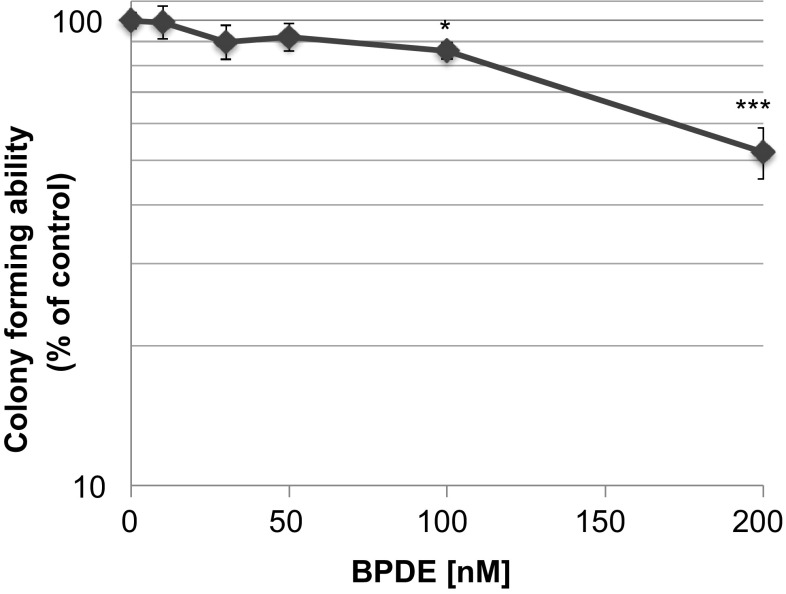
Cytotoxicity of BPDE determined via colony forming ability after 1 h treatment in TK6 cells. Shown are mean values of six determinations derived from two independent experiments ± SD. Statistically significant different from control: *p ≤ 0.05, ***p ≤ 0.001 (ANOVA-Dunnett’s *T* test)

In a first step, we investigated the cytotoxicity of BPDE in TK6 cells after 1 h treatment. Up to 100 nM BPDE, no or very slight cytotoxicity was observed, determined as colony forming ability. At 200 nM BPDE, a significant decrease to 50% of control occurred (Fig. 1). For subsequent experiments, the low, non-cytotoxic dose range up to 50 nM was applied for DNA adduct quantification and mutagenicity testing, while the whole concentration range was applied for gene expression profiling to ensure the detection of effects occurring in the non-cytotoxic range, but also those effects restricted to cytotoxic concentrations.

### Induction of DNA adducts by BPDE

Next, we investigated the induction of DNA adducts by BPDE after 1 h treatment. We applied a sensitive test system, based on the formation of stable adducts predominantly at the *N* ^2^-position of guanine and the subsequent release of tetrols after acid hydrolysis. The tetrols were very sensitively quantified by HPLC with fluorescence detection, allowing the quantification of as little as 1 adduct/10^8^ base pairs or about 60 adducts per cell, requiring 10–100 µg DNA (Schwerdtle et al. 2002).

**Fig. 2:**
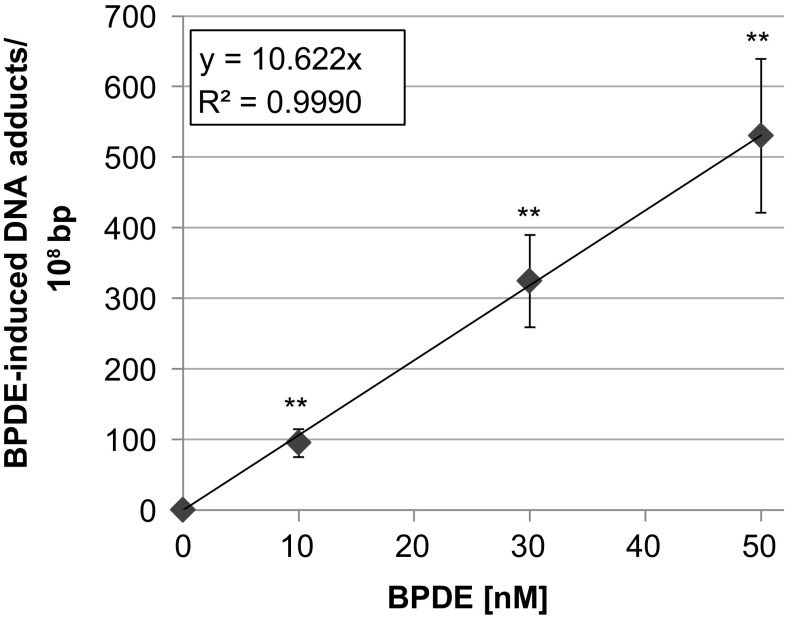
BPDE-induced DNA adduct levels determined via HPLC/FD after 1 h treatment in TK6 cells. Shown are mean values of four determinations derived from two independent experiments ± SD. Statistically significant different from control: **p ≤ 0.01 (ANOVA-Dunnett’s *T* test)

As shown in Fig. 2, a linear dose–response was observed in low dose range, yielding around 100 lesions/10^8^ base pairs at 10 nM and around 500 lesions/10^8^ base pairs at 50 nM BPDE.

### Induction of mutations by BPDE

**Fig. 3:**
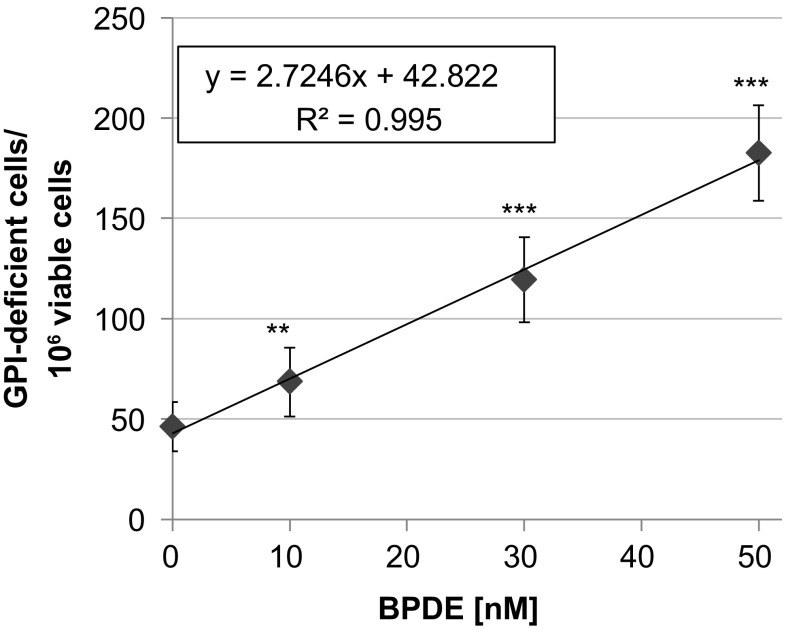
GPI(−) frequencies of TK6 cells after 1 h treatment with BPDE and 10 days of phenotype expression. Shown are mean values of 12 determinations derived from two independent experiments ± SD. Statistically significant different from control: **p ≤ 0.01, ***p ≤ 0.001 (ANOVA-Dunnett’s *T* test)

Subsequently, mutations were quantified by determination of GPI-deficient cells in the newly established in vitro PIG-A mutation assay. Within this test system, inactivating mutations in the *PIG-A* and in the *PIG-L* gene are phenotypically expressed by the loss of GPI-anchored proteins on the cell surface. The mutation frequency is equal to the frequency of GPI-deficient cells, which can be determined by antibody staining of GPI-anchored proteins and flow cytometry (Krüger et al. 2015, 2016). As shown in Fig. 3, significantly elevated levels of GPI-deficient cells were observed for the entire concentration range, starting at 10 nM BPDE and resulting in about 180 GPI-deficient cells per 10^6^ viable cells at 50 nM. Notably, mutations were induced in the completely non-cytotoxic concentration range of BPDE with respect to colony forming ability. As a parameter of cell growth after mutagen treatment, the relative increase in cell count (RICC) was determined, leading to a dose-dependent reduction down to about 55% at 50 nM BPDE (data not shown). All values were well above 10% as recommended by the OECD to exclude uninformative results in mutagenicity assays. Interestingly, the increase in mutation frequencies was linear with dose, starting at the lowest concentration.

### Correlation of DNA adducts and mutations

**Fig. 4:**
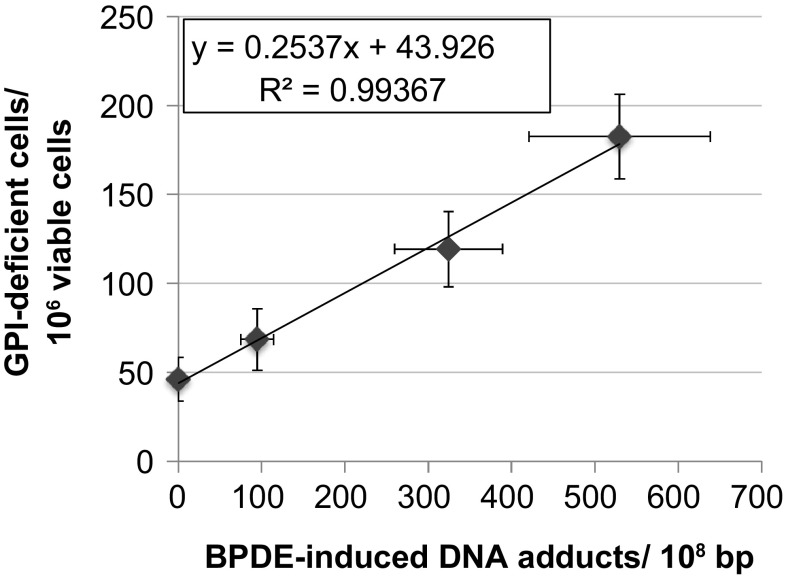
Correlation between GPI(−) frequencies with BPDE-induced DNA adduct levels in TK6 cells. Shown are mean values of 12 (GPI(−)) or four (DNA adducts) determinations derived from two independent experiments each ± SD

As stated in the introduction, one major question addressed in this study was whether or not there is a dose range in which DNA adducts are detectable but do not lead to the induction of mutations yet, perhaps due to efficient repair of DNA lesions in the low dose range. Therefore, mutation frequencies have been plotted against DNA adducts quantified by HPLC. As shown in Fig. 4, there was a linear correlation between the amount of DNA adducts and mutations in the entire concentration range of 0–50 nM BPDE.

### Repair of BPDE-induced DNA adducts

**Fig. 5:**
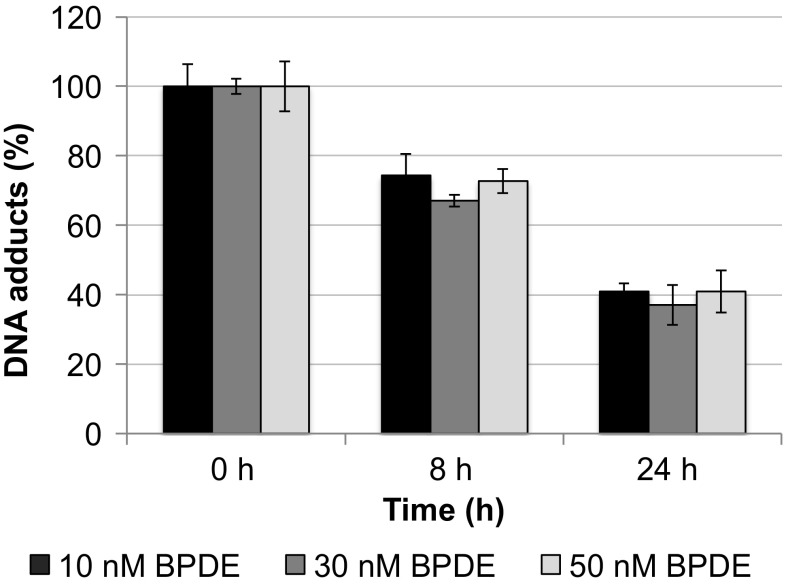
Time-dependent repair capacity of BPDE-induced DNA adducts in TK6 cells. For damage induction, cells were treated with the indicated BPDE concentrations for 1 h. Shown are mean values of four determinations derived from two independent experiments ± SD

As described above, BPDE-induced DNA adducts are removed via NER. However, actual repair capacities could vary between different cells lines. To exclude that the linear relationship between DNA adducts and mutations is due to missing repair in TK6 cells and to compare repair capacities at different treatment concentrations, we quantified DNA adduct levels after different post-incubation times. As shown in Fig. 5, around 30% of the adducts were removed within 8 h after treatment and around 60% after 24 h. First, this indicates that TK6 cells are repair proficient with respect to NER. Second, interestingly, the relative repair capacity was independent of the BPDE concentration, suggesting that repair efficiency is not increased at lower lesion density in the genome.

### Induction of the cellular response to BPDE-induced DNA damage by gene expression profiling

**Fig. 6:**
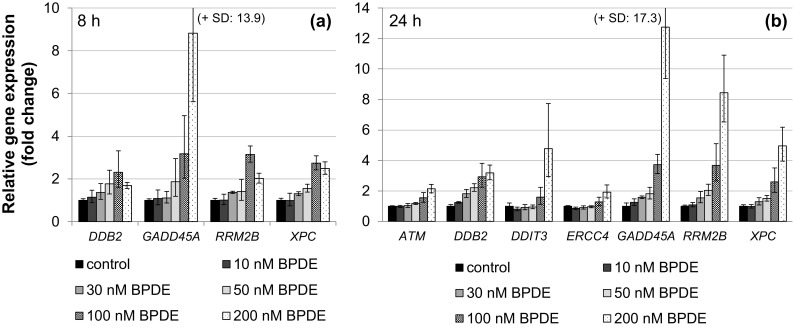
Impact of BPDE on gene expression related to DNA damage response and repair. TK6 cells were treated with BPDE for 1 h followed by 7 h (**a**) or 23 h (**b**) post-incubation, respectively. Shown are linear fold changes of the relative gene expression from mean values of four determinations derived from two independent experiments ± SD

**Fig. 7:**
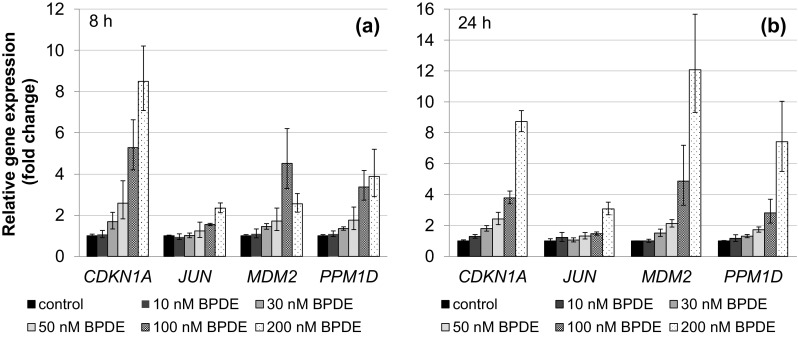
Impact of BPDE on gene expression related to p53 and AP-1 signaling. TK6 cells were treated with BPDE for 1 h followed by 7 h (**a**) or 23 h (**b**) post-incubation, respectively. Shown are linear fold changes of the relative gene expression from mean values of four determinations derived from two independent experiments ± SD

**Fig. 8:**
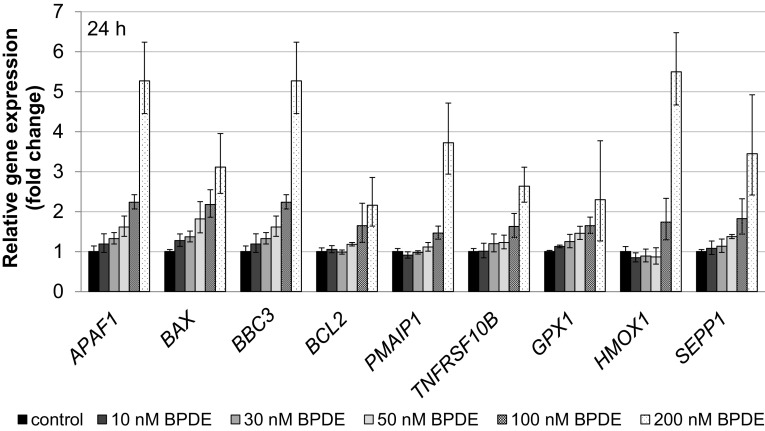
Impact of BPDE on gene expression related to oxidative stress response and apoptotic signaling. TK6 cells were treated with BPDE for 1 h followed by 23 h post-incubation. Shown are linear fold changes of the relative gene expression from mean values of four determinations derived from two independent experiments ± SD

To elucidate the cellular response to BPDE on the transcriptional level, we applied a recently established high-throughput RT-qPCR analyses with Fluidigm dynamic arrays using the BioMark™ System (Fischer et al. 2016). This method enables the parallel and quantitative analysis of expression levels of 95 different genes for 96 different samples. Genes were selected according to their relevance for genomic stability, comprising DNA damage response, DNA repair factors, oxidative stress response, cell cycle arrest, cell proliferation and apoptosis, and time- and concentration-dependent analyses were performed. Within the context of this study, one aspect of special interest concerned the question at which level of DNA lesions the DNA damage response would be activated on the transcriptional level. As shown in Fig. 6a, after 1 h incubation with BPDE and 7 h post-incubation, DNA damage signaling was evident most pronounced in case of *GADD45A*, followed by *RRM2B*. Also, the genes coding for the NER repair proteins DDB2 and XPC, both involved in DNA damage recognition, were induced. In each case, the effects were dose-dependent; however, relevant effects reaching twofold activation were restricted to concentrations of 100 nM BPDE and higher. After 23 h post-incubation, similar effects were observed; additionally, *ATM* involved in signaling and repair of DNA double strand breaks, *DDIT3* as a further DNA damage inducible gene as well as *ERCC4* coding for a NER nuclease were slightly induced (Fig. 6b). Likewise, p53- and AP-1 dependent signaling was observed in a concentration-dependent manner, again with pronounced effects mainly restricted to high BPDE concentrations (Fig. 7). Regarding p53-induced target genes, *CDKN1A* coding for the cell cycle inhibitor p21, the p53 inhibitor *MDM2* as well as the p53-inducible phosphatase *PPM1D* displayed distinct inductions at 100 nM BPDE and higher; in some cases, slight effects occurred already at 50 nM. The effects were more pronounced after 23 h (Fig. 7b) as compared to 7 h post-incubation (Fig. 7a). The positively auto-regulated *JUN* expression indicated AP-1 activation, with a relevant enhanced transcription restricted to 200 nM BPDE at both time points. As shown in Fig. 8, a similar dose–response relationship was evident with respect to the oxidative stress response as well as apoptotic signaling, exerting a clearly pro-apoptotic pattern after 1 h incubation followed by 23 h post-incubation. Thus, up to 50 nM BPDE no relevant modulation of gene expression was observed, whereas a more than twofold induction of the pro-apoptotic genes *APAF1, BAX,* and *BBC3* coding for the p53 up-regulated modulator of apoptosis (Puma), all involved in the intrinsic apoptotic pathway, was evident at 100 nM BPDE and higher. Increased mRNA levels of the pro-apoptotic *PMAIP1* coding for Noxa and *TNFRSF10B* activating the extrinsic apoptotic pathway, as well as the anti-apoptotic *BCL2* were even limited to 200 nM BPDE. Since *BAX, BBC3*, *PMAIP1,* and *BCL2* are known p53 target genes, this tumor suppressor appears to be activated at 100 nM BPDE and higher. Similarly, the transcriptional oxidative stress response as evident by the induction of the ROS-inducible *HMOX1* together with *GPX1* and *SEPP1* as anti-oxidative factors occurred only at the highest concentration of 200 nM BPDE. The complete gene expression profile is provided as a heat map in Supplementary Fig. 1.

## Discussion

One critical aspect discussed within the context of risk assessment for chemical carcinogens consists in the question whether there is a general “threshold” between the induction of DNA adducts and their conversion into mutations, potentially mediated by efficient repair in case of only few adducts and/or protective adaptive mechanisms within the transcriptional response to DNA damage. This is of particular interest in case of low, for human exposure relevant levels. As stated in the introduction, there have been numerous contributions stating that for alkylating agents there may be a dose range where DNA adducts are measurable but do not result in increased mutation frequencies; for recent review see (Klapacz et al. 2016). Nevertheless, this raises the question whether this is a general phenomenon or restricted to certain DNA lesions.

Within the present study, we selected BPDE, the critical DNA reactive metabolite of BaP, which provokes helix distorting lesions in DNA repaired by NER, to address the question whether there is a concentration range where DNA adducts occur without measurable increase in mutation frequencies. We quantified stable DNA adducts at the *N* ^2^-position of guanine, mutations by the PIG-A assay, as well as the transcriptional response to DNA damage via a high-throughput qRT-PCR technique. Important prerequisites to approach this question were comparable incubation conditions, including the identical cell line, in this case TK6 cells, expressing the GPI anchored proteins used as marker proteins for mutagenicity. Also, with regard to the different endpoints, similar detection sensitivities for the low dose range were of major importance, to exclude that a potential restriction of mutations to higher concentrations results from higher detection limits as compared to DNA adduct levels.

Regarding the quantification of DNA lesions, BPDE-DNA adduct levels were measured by a highly sensitive HPLC/fluorescence assay described previously (Schwerdtle et al. 2002). The principle of the procedure consists in the hydrolysis of stable DNA adducts formed at the *N* ^2^ position of guanine by 0.1 N HCl, yielding the corresponding tetrol I-1 (Rojas et al. 1994). Under our experimental conditions, the assay exerts a detection limit of 1 pg of tetrol I-1, requires 10–100 µg DNA and detects 1 adduct/10^8^ base pairs or about 60 adducts/cell. This high sensitivity allows the reproducible quantification of adduct formation and repair after incubation with low, non-cytotoxic concentrations of BPDE. Within the present study, significant increases in lesions were detected at concentrations as low as 10 nM, with a linear increase up to 50 nM BPDE. For comparison, immunological detection required 25-fold higher concentrations (0.25 µM and above) (Christmann et al. 2016). Regarding the repair of the adducts, only 30% were removed within 8 h post-incubation time, and 40% were still left after 24 h. This repair course is in agreement with previous observations in A549 and HCT116 cells (Grosskopf et al. 2010; Piberger et al. 2014; Schwerdtle et al. 2002), supporting similar repair capacities in different cell lines. Interestingly, repair kinetics were independent of the applied dose, indicating that within this low dose range of BPDE, no differences in relative repair were observed, and thus, no dose showed faster or even complete repair. This may be explained by specific features of NER mediating the repair of stable BPDE-DNA adducts. NER removes structurally unrelated bulky base adducts generating significant helical distortions. According to the current knowledge, it involves at least 30 different proteins and enzymes in mammalian cells, including those which are defective in patients suffering from the DNA repair disorder Xeroderma Pigmentosum (XP) complementation groups A through G (de Boer and Hoeijmakers 2000). Two different pathways can be distinguished: the global genome repair (GG-NER) operating in all parts of the genome and the transcription-coupled repair (TC-NER) eliminating DNA damage from the transcribed strand of active genes. While TC-NER is usually fast and efficient to restore transcription, GG-NER on the other hand is slower and may be incomplete, leading to an accumulation of mutations in poorly repaired regions (Fousteri and Mullenders 2008; Mullenders et al. 1991). Accordingly, three levels of repair efficiencies have been identified in human fibroblasts after treatment with BPDE: The transcribed strand of the active *HPRT* gene was repaired about twice as fast as the non-transcribed strand, resulting in 53 and 26% of adduct removal after 7 h, respectively. In contrast, only 14% of BPDE adducts were removed from the inactive locus *754* within 20 h (Chen et al. 1992). In addition, the rates of incision of stereochemically identical BPDE-induced DNA lesions catalyzed by the prokaryotic UvrABC system was shown to be higher in the TG*T than in the CG*C sequence context (Ruan et al. 2007), due to differences in structural alterations of the DNA helix (Cai et al. 2007). The longevity of at least some PAH-induced DNA adducts was also shown in lung autopsy samples of non-smokers, ex-smokers, and smokers. Lowest frequencies of lesions were found in the first group, intermediate frequencies in the second, and highest values in the third group. Furthermore, almost all samples even of the non-smoking group had detectable levels of PAH-induced DNA lesions, indicating that even low levels of environmental exposure lead to unrepaired DNA adducts (Lodovici et al. 1998).

In this study, mutations induced by BPDE were quantified by the recently established PIG-A assay. This approach is based on the phenotypic alteration of the GPI status by mutations diminishing the GPI biosynthesis, which can be measured via multicolor flow cytometry with an antibody staining protocol of GPI-anchored proteins CD55 and CD59. In addition to the X-chromosomal *PIG*-*A* gene, TK6 cells have been shown to additionally harbour a heterozygous deletion in the *PIG*-*L* gene. Since both gene products are required for GPI biosynthesis, there are two reporter genes leading to the same phenotypic alteration, rendering this test system particularly sensitive towards both point mutations and deletions, also when compared to the HPRT assay (Krüger et al. 2015, 2016). Previously, we demonstrated the dose-dependent, statistically significant mutagenicity of ethyl methanesulfonate (EMS), UV-C irradiation, and 4-nitrochinoline-*N*-oxid (NQO), while pyridine and cycloheximide, applied as non-mutagenic negative controls, were also correctly identified as non-mutagens (Krüger et al. 2015). Within the present study, BPDE increased the level of GPI-deficient mutant cells in a dose-dependent manner, with no obvious deviation from linearity also at the lowest concentrations. Furthermore, there was a linear correlation between DNA adduct formation and mutagenicity, again arguing against a “no effect” range in the low dose exposure towards BPDE.

The transcriptional response to BaP or BPDE-induced DNA damage has been investigated previously, for example via microarray analysis to elucidate the impact of p53 (Hockley et al. 2008). Also, recently, the adaptive up-regulation of selected DNA repair genes was analyzed and correlated to BPDE-induced DNA adduct levels, showing that transcriptional activation protects against BPDE-induced cytotoxicity at the expense of mutations; the latter due to the enhanced expression of error-tolerating POLH (Christmann et al. 2016). Nevertheless, to the best of our knowledge, no gene expression analysis study was conducted in the very low concentration range of 10–200 nM BPDE as it is presented within this study. Furthermore, we applied a high-throughput RT-qPCR technique, enabling quantitative time- and concentration-dependent gene expression analyses for 96 samples in parallel, providing a comprehensive gene expression profile with respect to DNA damage response, DNA repair factors, oxidative stress response, cell cycle arrest, cell proliferation, and apoptosis. Special focus was given to time- and concentration-dependent inductions of gene expression coding for representative factors of the above-mentioned pathways with special relevance for genomic stability, investigated under the same incubation conditions as used for DNA adduct and mutation quantifications. As expected, treatment with BPDE induced genes coding for DNA damage signaling such as *GADD45A*, DNA repair factors involved in DNA damage recognition during NER, p53 and AP-1 dependent signaling, as well as those coding for oxidative stress response and pro-apoptotic factors. However, almost all significant changes in gene expression were restricted to the two highest concentrations applied, 100 and 200 nM BPDE, while highly significant increases in mutation frequencies were observed at concentration levels 10- and 20-fold lower. Therefore, neither the induction of DNA repair genes nor, for example, p53-dependent cell cycle control or apoptotic genes were able to protect against BPDE-induced mutations in the very low dose range.

This raises the question how BPDE-induced DNA adducts are converted into mutations. BPDE-induced DNA adducts impair DNA synthesis as well as global replication fork progression, and two mechanisms appear to be associated with overcoming stalled replication fork upon BPDE-induced DNA adducts. One consists in lesion bypass by mainly error-prone DNA translesion synthesis (TLS) polymerases. This process is thought to be critical for BaP-caused mutagenicity via the induction of point mutations and has been extensively studied (Bi et al. 2005; Lagerqvist et al. 2011; Temviriyanukul et al. 2012). Additionally, stalled or damaged replication forks may be restarted or repaired via homology-dependent mechanisms (Cohen et al. 2015; Dumstorf et al. 2009; Izhar et al. 2013; Langie et al. 2007). Even though homologous recombination (HR) and HR-dependent mechanisms are intrinsically error-free, recombination could give rise to gross chromosomal instabilities such as genomic rearrangements or loss of heterozygosity when promoted by micro-homology or any other homologous sequence near the fork (Carr and Lambert 2013). Further investigations are needed to clarify whether mutations induced in response to low dose of BPDE as applied in the present study are exclusively mediated via error-prone TLS or might be in part induced by HR events as well.

### Conclusions and outlook

Our study seems to contradict assumptions of a general “biological threshold” for genotoxic carcinogens. Thus, in case of BPDE-induced DNA adducts, both DNA lesion induction, as well as mutagenicity followed linear dose–response relationships in the very low dose range, and were highly correlated. In contrast, the transcriptional response to DNA damage was restricted to higher, partly cytotoxic concentrations. One reason for the discrepancy between the linear correlation of DNA damage and mutations shown here and the reported “threshold” in response to some alkylating agents appears to be the impact of different types of DNA lesions, especially also different types of DNA repair systems involved in their removal. Without any doubt, NER is a largely error-free process, which protects from mutagenicity, including BPDE-induced DNA adducts (Lagerqvist et al. 2011). Nevertheless, NER may be less effective as compared to BER involved in the removal of different DNA lesions induced by alkylating agents. While the latter repair system removes DNA lesions that also occur endogenously, nucleotide excision repair is involved in the removal of bulky lesions, frequently induced by environmental carcinogens. As described above and shown within the present study, NER has been shown to be slower and less effective, due to heterogeneity of repair throughout the genome and also with respect to the DNA sequence in which the lesion is located. Nevertheless, even though DNA repair capacities were found to be similar in different cells lines, they may differ in vivo in different tissues; this needs to be further investigated. This aspect also accounts for the transcriptional response to DNA damage. Altogether, our results indicate that the question of a “threshold” between the induction of DNA lesions and their conversion into mutations needs to be considered on a case-by-case basis, taking into account different DNA repair systems and respective lesions. Whether or not our observations derived for treatment with BPDE also apply to other substrates of NER requires future research.

## Electronic supplementary material

Below is the link to the electronic supplementary material.

**Supplementary Fig.** **1: Impact of BPDE on gene expression related to oxidative stress response, apoptosis, cell cycle arrest and proliferation, transcription factors and DNA damage response and DNA repair.** TK6 cells were treated with BPDE for 1 h followed by 23 h post-incubation. Shown are linear fold changes of the relative gene expression from mean values of four determinations derived from two independent experiments ± SD. (PPTX 94 kb)
