# Supplementary material for: BPDE-induced genotoxicity: relationship between DNA adducts, mutagenicity in the in vitro PIG-A assay, and the transcriptional response to DNA damage in TK6 cells
Source: Arch Toxicol. 2017 Jun 7;92(1):541–51. doi: 10.1007/s00204-017-2003-0 (PMC5773665; doi:10.1007/s00204-017-2003-0)
Supplement: Supplementary file 1 — Supplementary Fig. 1: Impact of BPDE on gene expression related to oxidative stress response, apoptosis, cell cycle arrest and proliferation, transcription factors and DNA damage response and DNA repair. TK6 cells were treated with BPDE for 1 h followed by 23 h post-incubation. Shown are linear fold changes of the relative gene expression from mean values of four determinations derived from two independent experiments ± SD. (PPTX 94 kb) [file 204_2017_2003_MOESM1_ESM.pptx]

## Slide 1
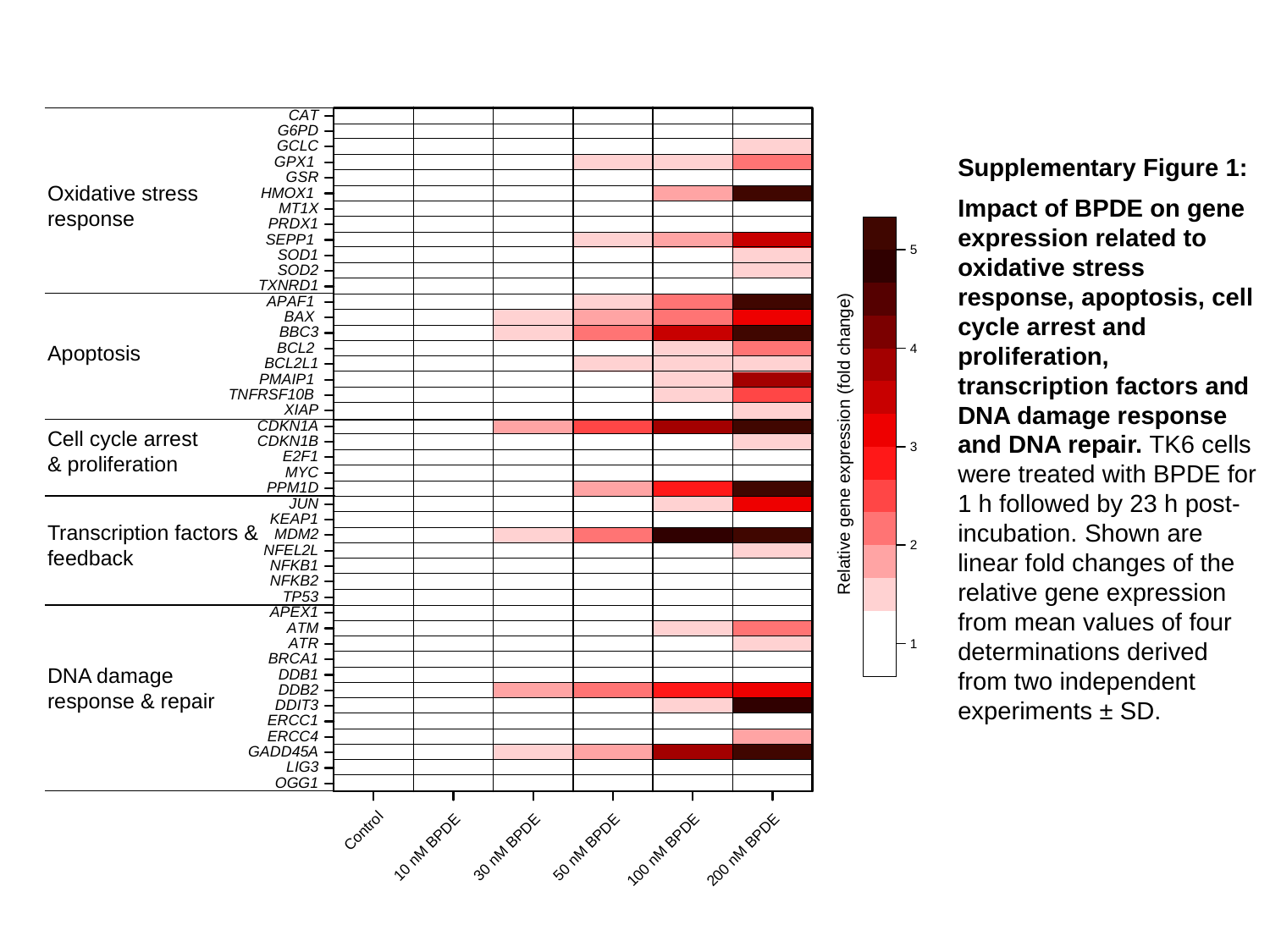

Oxidative stress response
Apoptosis
Cell cycle arrest
& proliferation
Transcription factors & feedback
DNA damage response & repair
Supplementary Figure 1:
Impact of BPDE on gene expression related to oxidative stress response, apoptosis, cell cycle arrest and proliferation, transcription factors and DNA damage response and DNA repair. TK6 cells were treated with BPDE for 1 h followed by 23 h post-incubation. Shown are linear fold changes of the relative gene expression from mean values of four determinations derived from two independent experiments ± SD.
